# A systematic review of case reports of hepatic actinomycosis

**DOI:** 10.1186/s13023-021-01821-5

**Published:** 2021-04-30

**Authors:** Zahra Chegini, Mojtaba Didehdar, Seidamir Pasha Tabaeian, Amin Khoshbayan, Aref Shariati

**Affiliations:** 1 Department of Microbiology, School of Medicine, Iran University of Medical Sciences, Tehran, Iran; 2 Department of Medical Parasitology and Mycology, Arak University of Medical Sciences, Arak, Iran; 3 Department of Internal Medicine, School of Medicine, Iran University of Medical Sciences, Tehran, Iran; 4 Colorectal Research Center, Iran University of Medical Sciences, Tehran, Iran

**Keywords:** Actinomycosis, Liver abscess, Hepatic actinomycosis, *Actinomyces* species, Diagnosis

## Abstract

**Background:**

Hepatic Actinomycosis (HA) is one of the infections that causes disorders in patients when diagnosed untimely and inappropriately.

**Methods:**

Case reports on HA in patients published between 2000 and April 2020 were gathered by carrying out a structured search through PubMed/Medline.

**Results:**

Through a survey of the Medline database, 130 studies were identified and then, 64 cases with HA were included in the final analysis. Asia had the largest share of cases with 37.5% (24 reports), followed by Europe and the Americas. Affected patients were predominantly males (64%) and the overall mortality rate was 1% with only one male patient in his 50 s dying. Nearly all patients (92%) were immunocompetent. However, in four patients, the use of immunosuppressive medication led to depression of the immune system. Most of the patients (80%) experienced complications. In terms of the complications, the most frequent ones were previous history of abdominal surgery (32%) and foreign bodies in the abdominopelvic region (20%). *Actinomyces israelii* was the most common pathogen isolated from patients. Abdominal pain (66%), fever (62%), weight loss (48%), night sweat, malaise, and anorexia (14%) over about 3.1 months were the most frequently reported clinical symptoms. Extension to one or more surrounding organs was evident in 18 patients (28%). Histopathologic examination confirmed infection in 67% of the patients and samples obtained from liver puncture biopsy (32%) were most frequently used in diagnosis. Surgery or puncture drainage + anti-infection was the most common method to treat patients and penicillin, Amoxicillin, Doxycycline, and ampicillin were the most frequently used drugs to control infection.

**Conclusion:**

HA should be considered in patients with a subacute or chronic inflammatory process of the liver. With accurate and timely diagnosis of infection, extensive surgery can be prevented.

**Supplementary Information:**

The online version contains supplementary material available at 10.1186/s13023-021-01821-5.

## Introduction

*Actinomyces* spp. are opportunistic gram-positive, anaerobic, or facultatively anaerobic bacteria that usually colonize the upper respiratory tract or the gastrointestinal and female genital tract and typically infect males between 20 and 60 years old [1, 2]. Granulomatous inflammation, abscesses, contiguous spread, and formation of sinus tract fistulae are the distinguishing characteristics of chronic actinomycosis. This type of infection has been addressed in the research for more than 150 years with the most common agent being *Actinomyces israelii* [2–4]. *Actinomyces* have low pathogenicity and their infection has subacute manifestation. However, polymicrobial infections frequently occur in patients being involved with actinomycosis, promoting pathogenicity of these bacteria [3, 5]. Fortunately, drug resistance is not an issue in the treatment procedure and beta-lactams, particularly penicillin G or Amoxicillin, are extremely influential in *Actinomyces* spp., making them an ideal choice for treatment [6, 7]. Treatment of actinomycosis should focus on antibiotic therapy and surgery alone does not seem to be a suitable method for treatment, although in refractory cases, surgery can be a supporting therapy [3, 8].

While any site in body can be involved with actinomycosis, cervicofacial infection is the most common one. Other parts of the body prone to be infected with *Actinomyces* spp. are thorax, abdomen, pelvis, and the central nervous system [3, 4]. Abdominal infection occurs in 20% of cases and appendix and the ileocecal region are generally affected [9]. Hepatic involvement is usually uncommon and occurs after other intra-abdominal sites of infection. Hepatic Actinomycosis (HA) occurs in only 15% of the abdominal infections and, in total, makes up 5% of all actinomycosis cases [5, 10]. Diagnosis of HA is often challenging, because infection diagnosis is dependent on clinical manifestations and findings from imaging. Thus, it can be simply misdiagnosed as a primary liver cancer when it is in the form of a solitary tumor or metastatic with multiple imaging features [8, 11, 12]. In this regard, actinomycosis often mimics malignancy, tuberculosis, or nocardiosis due to its progressive and constant expansion that may form a cold abscess [7, 13]. Therefore, diagnosing HA in patients is usually challenging and in some cases, this leads to extensive surgery and permanent damage. The present research is the first systematic review aimed at studying the reported HA cases in different patients.

## Method

### Literature search and inclusion criteria

**Fig. 1:**
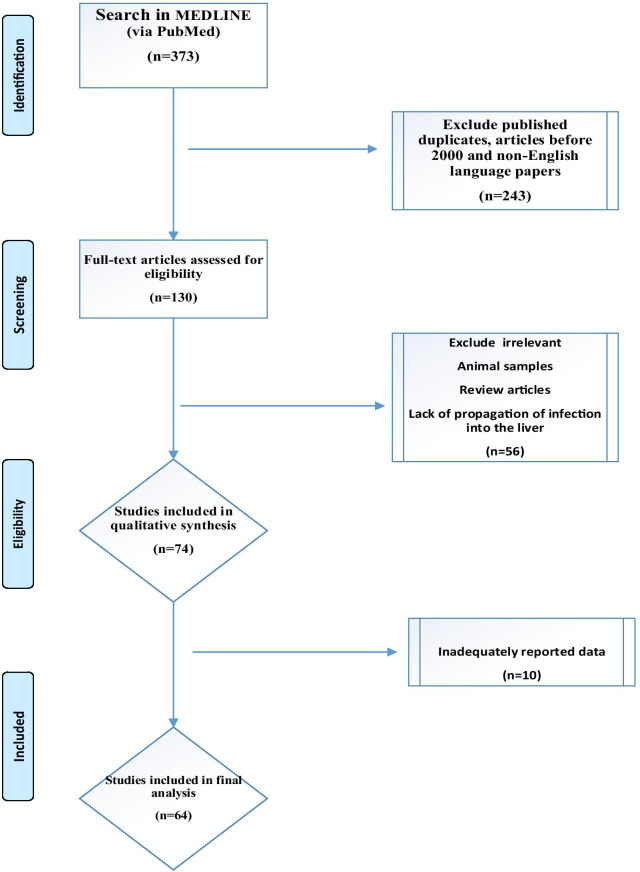
Flow chart of publication selection and their inclusion in the systematic review

In the present research, a Medline (via PubMed) search was carried out between the years 2000 and 2020. The keywords for search were taken from the National Library of Medicine’s Medical Subject Heading (MeSH) terms, titles, or abstracts through Boolean operators (and/or) including “*Actinomyces*” or “Actinomycosis” or “Actinomycotic”, and “Liver” or “Hepatic.” It should be noted that only studies in English language were included. Of the total 130 hits, 64 were included based on the inclusion criteria given in Fig. 1. The protocol for review employed in the present research was adopted from our recent article and a study conducted by Hickey et al. [14, 15].

### Inclusion criteria

The systematic review was carried out by the given inclusion criteria: individual case reports on HA in different patients, full texts or abstracts of studies published in English, and online studies published on Medline (via PubMed) from 2000 up to April, 2020.

### Exclusion criteria

The exclusion criteria were non-human studies, review articles, guidelines, systematic reviews or meta-analyses, actinomycosis without propagation of infection into the liver, and incomplete reported data (Fig. 1).

### Study selection and data extraction

Two researchers (AS and ZC) screened the articles and when discrepancy was observed, they skimmed the paper or conference abstract as a case to be reviewed by both in collaboration. For each article, the following features were extracted and recorded through Excel software (Microsoft, Redmond, WA, USA): country, publication year, sex, age, causative pathogen, medical history and co-morbidities, clinical presentation, antibiotics treatment, surgery and drainage, diagnostic method, and outcome. The references of the articles were comprehensively surveyed to make sure that there were no additional cases remaining unidentified from the primary search.

### Quality assessment

The critical checklist for appraisal put forward by the Joanna Briggs Institute (JBI) was adopted in order to assess the quality of studies [16].

## Results

### Epidemiology

In a survey of the Medline database, 130 cases were identified and then, 64 patients with HA were included in the final analysis based on the study criteria (Fig. 1). These individual cases were published from Bahrain, India, France, Italy and Portugal (one each), Canada, Germany, Greece, Romania, Spain, and Serbia (two each), the United Kingdom (three reports), Japan (four reports), China (five reports), Taiwan and Turkey (six each), Korea (seven reports) and finally the USA (16 reports). Accordingly, Asia had the largest share of cases with 37.5% (24 reports) followed by Europe and the Americas with 34% (22 reports) and 28% (18 reports), respectively. No case from the continents of Australia and Africa was detected. According to the findings of the present study, the overall mortality rate was 1% with only one male patient in his 50 s dying due to thoraco-pulmonary and HA condition that rapidly deteriorated and killed him before starting treatment. Our results also showed that only 36% of the patients were female and the other 64% were male. The mean age of patients was 51.6 years (ranging between 5 and 84) and 55% of the patients were 50 years of age or older (Table 1).

**Table 1:** Clinical, diagnosis, epidemiological and therapeutic features of patients with hepatic actinomycosis from individual cases

Country, year of publication and references	Sex/age	Species	Treatment	Surgery or puncture drainage	Diagnosis
Bahrain, 2020 [17]	49/f	NR	Ampicillin discontinued due to sepsis, Doxycycline for 6 month	Thoracentesis and insertion of a chest drain	HE
Canada, 2001 [18]	50/m	NR	Penicillin	NR	HE
Canada, 2005 [19]	40/F	NR	Penicillin G	Abdominal hysterectomy and bilateral oophorectomy	HE and culture
China, 2004 [20]	64/m	NR	Amoxicillin/clavulanic acid for 6 months	Left lateral segmentectomy and distal gastrectomy were performed	HE
China, 2011 [21]	47/F	*A. israelii*	Ampicillin for 4 weeks, followed by oral Amoxicillin for 2 months	Lobectomy of the left liver lobe, A right oophorectomy	HE
China, 2013 [22]	67/M	NR	Penicillin G and oral penicillin for six month	Left lateral hepatectomy and distal gastrectomy	HE
China, 2014 [12]	55/M	NR	Penicillin	Left lobe resection	HE
China, 2016 [23]	38/M	NR	Cefoperazone for 7 days after the surgery	Left lobectomy of the liver	HE
France, 2013 [24]	34/M	NR	Ampicillin, Penicillin G	Appendectomy	HE
Germany, 2009 [25]	71/f	NR	Amoxicillin, imipenem/cilastatin for *Klebsiella pneumonia*	NR	HE
Germany,2001 [26]	57/m	NR	Penicillin G then Clindamycin for six month	NR	HE
Greece, 2004 [27]	53/M	NR	Ciprofloxacin for 6 weeks	A right posterior segmentectomy of the liver was performed	HE
Greece, 2010 [28]	70/M	NR	Amoxicillin-sulbactam, amoxicillin	NR	HE
India, 2005 [29]	35/m	NR	Penicillin G, ampicillin for 5 months	Ultrasound-guided percutaneous aspiration of the liver abscess	Culture
Italy, 2005 [30]	31/M	NR	Piperacillin	Surgical resection of the IV_/VII hepatic segments	HE with a positive culture
Japan, 2011 [31]	74/F	*A. israelii*	Ampicillin, Amoxicillin for 6 month	Aspiration drainage and placed an indwelling catheter immediately	PCR and culture
Japan, 2012 [9]	60/M	NR	Sulbactam/ampicillin for 36 days, sultamicillin for six month	Video-assisted thoracic surgery	HE
Japan, 2014 [32]	80/M	NR	Ampicillin/sulbactam, erythromycin, Ampicillin/sulbactam for two years	A drainage tube was inserted into the right thoracic space	HE
Japan, 2016 [33]	51/F	NR	Levofloxacin and warfarin treatment	Right salpingo-oophorectomy	HE, negative culture
Kore, 2006 [34]	47/F	NR	Penicillin G, Amoxicillin for 5 months	Left salpingo-oophorectomy, abdominal hysterectomy, right salpingo-oophorectomy	HE
Korea, 2012 [35]	72/M	NR	Penicillin for two month	Percutaneous drainage for 2 weeks	HE
Korea, 2012 [36]	41/f	NR	Ampicillin, amoxicillin treatment for four months	A right salpingo-oophorectomy with left ovarian excision and IUD removal was carried out	HE
Korea, 2012 [37]	67/m	*A. cardiffensis*	Ceftriaxone, Amoxicillin for 6 months	NR	Culture, PCR
Korea, 2013 [8]	57/M	NR	Penicillin G, ceftriaxone, Amoxicillin for 11 weeks	Exploratory laparotomy	HE
Korea, 2018 [38]	55/F	NR	Penicillin for 6 months	Bilateral salpingo-oophorectomy, small bowel resection and appendectomy	HE
Korea, 2018 [39]	67/M	NR	NR	Lobectomy of the liver	HE
Portugal, 2014 [4]	38/M	NR	Doxycycline for three months	Left hepatectomy extended to segments V and VIII	HE
Romania, 2012 [40]	72/f	NR	Ampicillin for two months, then Doxycycline for 139 days	Surgical drainage	HE
Romania, 2013 [41]	54/m	NR	Broad-spectrum antibiotics and antimycotics	Laparoscopy and biopsy	HE
Serbia, 2009 [42]	50/F	NR	Benzylpenicillin for 6 weeks and oral Amoxycillin for 6 months	Affected liver segments were resected	HE
Serbia, 2018 [43]	50/F	*NR*	Amoxicillin during three months	Liver abscess resected, hysterectomy, extraction of the IUCD	HE
Spain, 2011 [44]	20/m	*A. israelii*	Amoxicillin–clavulanate for two month	CT-guided pericardial drainage and A left-sided thoracocentesis, Pericardial and hepatic drainages	Culture and PCR
Spain, 2017 [45]	66/m	*A. naeslundii*	Ertapenem for 4 weeks	Percutaneous drainage of the abscess	Culture
Taiwan, 2001 [46]	6/m	NR	Penicillin then Clindamycin for 4 month	The right kidney and liver were adequately debrided	HE
Taiwan, 2005 [47]	71/m	NR	Penicillin G and V for 3 month	NR	HE
Taiwan, 2005 [47]	73/f	NR	Penicillin V for 3 month	Segmentectomy	HE
Taiwan, 2010 [48]	78/m	*A. odontolyticus*	Ceftriaxone, ampicillin–sulbactam and Amoxicillin for 6 weeks	CT-guided pigtail catheter insertion for drainage of the abscess	Culture, PCR
Taiwan, 2013 [49]	49/f	*A. israelii*	Benzyl penicillin and imipenem/cilastatin	An exploratory laparotomy and right lobectomy of the liver	HE
Taiwan,2009 [1]	37/m	NR	Penicillin for 3 months	Resected spleen and liver nodules	HE
Turkey 2010 [50]	40/M	NR	Penicillin G for 2 month	Surgical debridement and drainage was performed	HE
Turkey, 2002 [51]	41/F	NR	Penicillin G	The mass was excised	HE
Turkey, 2003 [52]	11/F	NR	Penicillin G for 1 month then Amoxicillin	NR	Culture
Turkey, 2006 [53]	40/F	NR	Penicillin G, a 6-month amoxicillin	Thoracentesis and thorax tube was inserted upon the diagnosis of empyema	HE with negative culture
Turkey, 2007 [54]	5/F	NR	Penicillin G for the next 6 months	NR	HE
Turkey, 2007 [55]	46/m	NR	Penicillin G 3 month	NR	HE
UK, 2009 [2]	52/M	NR	Teicoplanin, meropenem and metronidazole then six month amoxicillin	The hepatic abscess was aspirated and a drain, anterior colonic resection with primary anastamosis	Culture
UK, 2011 [56]	50/M	NR	Died before treatment	NR	Autopsy (The patient died)
UK, 2012 [57]	38/f	*A. israelii*	co-amoxiclav for 7 month	Ultrasound-guided drainage of the liver abscess was required 5 months later due, in part, to poor compliance with antibiotic treatment	HE, culture
USA, 2000 [58]	40/f	*A. israelii*	Penicillin	NR	Gram and immunofluorescent staining
USA, 2001 [59]	16/m	NR	Penicillin 6 months oral doxycycline	Laparotomy and open biopsy	HE and culture
USA, 2002 [60]	59/M	*A. turicensis*	Piperacillin-tazobactam, Doxycycline orally for 4 months	The abscess was aspirated and a drain was placed	Culture and PCR
USA, 2002 [61]	34/m	NR	Ciprofloxacin was continued for 6 weeks and Clindamycin for 3 months	The abscess was incised and drained	Gram staining
USA, 2005 [62]	84/f	NR	Penicillin	NR	Gram staining
USA, 2006 [5]	46/m	*A. israelii*	Penicillin	Percutaneous aspiration of the two largest liver collections	Culture
USA, 2006 [5]	59/m	*A. israelii*	Penicillin	Drainage	Culture
USA, 2010 [10]	75/F	NR	Clindamycin for 6 months	Laparotomy with biopsy of the liver	HE
USA, 2011 [63]	65/m	NR	Doxycycline for 6 month	Resection of segment 5 and 6 of the liver without any complications	Culture
USA, 2013 [64]	48/m	*A. naeslundii*	Amoxicillin-clavulanate for six month	Drainage catheter	Culture
USA, 2016 [65]	43/M	NR	Penicillin G for one month then Amoxicillin for one year	Right hemi hepatectomy	HE
USA, 2016 [66]	73/m	NR	Penicillin G	Incision and drainage of an abscess	HE
USA, 2017 [67]	70/m	NR	Penicillin	NR	HE
USA, 2017 [68]	80/m	NR	Penicillin G for 4 weeks then transitioned to oral penicillin for about 9 months	Aspiration of the fluid collection	HE and culture
USA, 2019 [69]	35/F	NR	Ampicillin /sulbactam for 6 weeks	NR	HE
USA, 2020 [70]	59/M	A. naeslundii or A. viscosus	Amoxicillin for 6 month	Extraction of a hepatic segment II	PCR

*M* Male, *F* Female, *HE* histopathological examination, *NR* not reported, *PCR* polymerase chain reaction

**Fig. 2:**
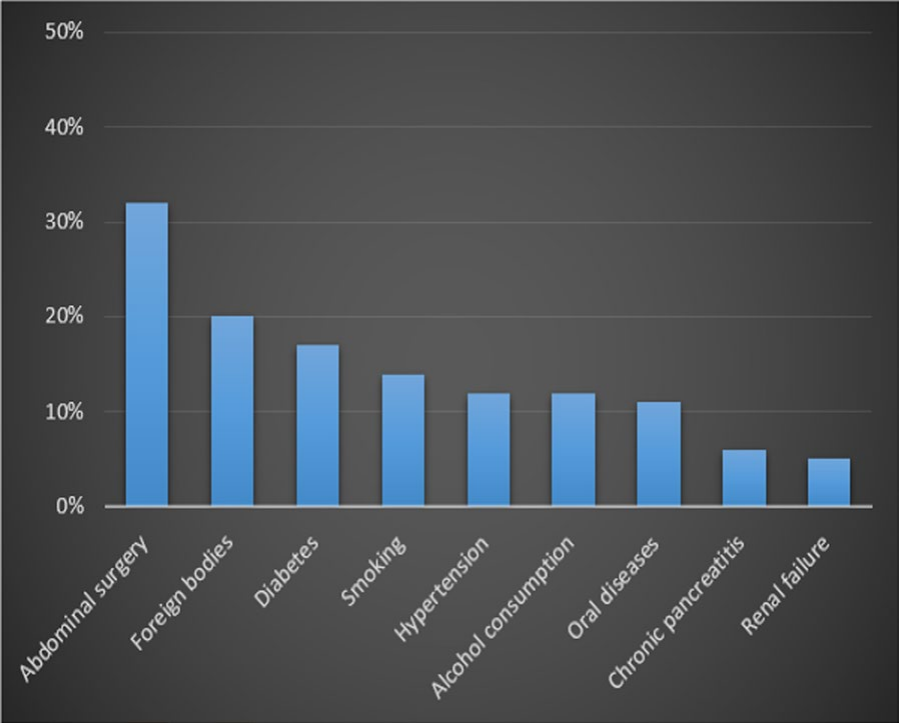
The most common complications in patients with hepatic actinomycosis

Primary or isolated HA (when a primary focus was not found) was reported in 61% of the patients, while in 20 (31%) and 4 (6%) patients, abdominopelvic actinomycosis with liver dissemination and disseminated infection were reported, respectively. Also, in one patient, actinomycosis of lungs directly invaded the liver parenchyma with IgG4-related hepatopathy through the diaphragm. Nearly all patients (92%) were immunocompetent; however, in four patients, the use of immunosuppressive medication prednisolone, plaquinil, prednisone, and tacrolimus led to depression of the immune system. In one patient, immunodepression by varicella was reported as the most likely reason for the acute onset of symptoms. On the other hand, most of the patients (80%) had complications with the most frequent one being previous history of abdominal surgery (32%), foreign bodies in the abdominopelvic region (20%) (Intrauterine Contraceptive Device (IUD) in nine cases, stenting of the bile duct in two cases, pancreatic duct stenting, and accidentally ingested fish bone) and diabetes (17%) (Fig. 2).

Only 14 studies (22%) performed the species-level identification and *A. israelii* was the most common pathogen isolated from patients with eight reports, followed by *A. naeslundii* with two reports and *A. cardiffensis*, *A. odontolyticus*, and *A. turicensis* each with one case. Notably, in one study, the causative pathogen was reported to be of either *A. naeslundii* or *A. viscosus* species considering the fact that there was no difference between the two in Polymerase Chain Reaction (PCR) with 16 s rRNA primer set (Table 1). Finally, the infection was mixed with other organisms in 17 cases (26%) with the available information in Additional file 1: Table S4.

### Clinical manifestations

**Fig. 3:**
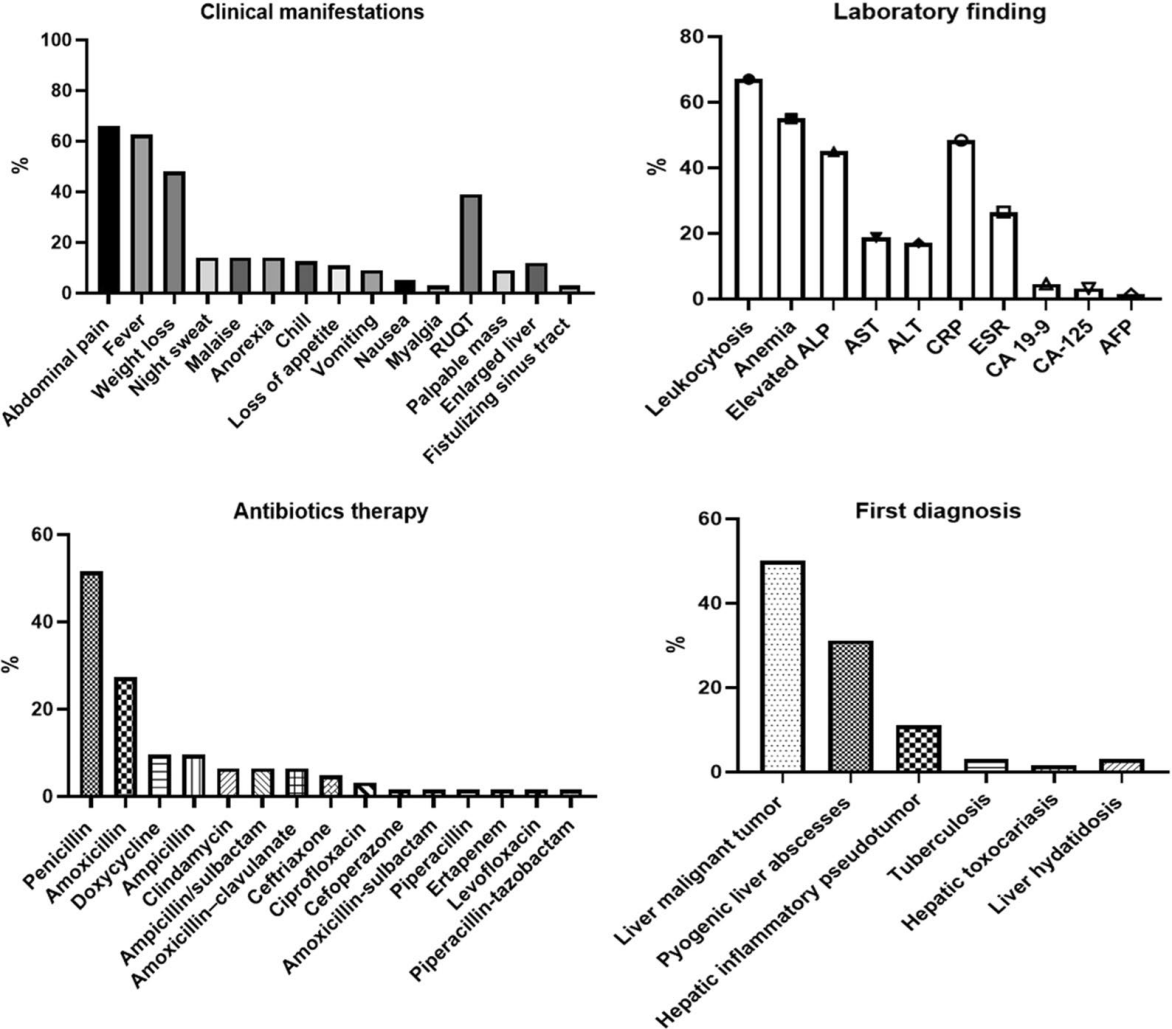
Characteristic of patients with hepatic actinomycosis. *RUQT* Right upper quadrant tenderness, *ALP* alkaline phosphatase, *AST* aspartate transaminase, *ALT* alanine transaminase, *CRP* C-reactive protein, *ESR* erythrocyte sedimentation rate, *CA* carbohydrate antigen, *AFP* alpha-fetoprotein

The most common manifestations were abdominal pain (66%), fever (62%) and weight loss (48%). Other clinical manifestations of patients are displayed in Fig. 3. Initial laboratory observations revealed leukocytosis, anemia, and elevated alkaline phosphatase (ALP) in 43 (67%), 35 (55%), and 29 (45%) patients, respectively (Fig. 3). Onset was chronic or subacute with an extended duration of symptoms (mean ± SD = 3.1 ± 3.9 month) such that the shortest duration was one month and the longest 2 years. A more acute presentation (< 2 week) was reported in 15 (26%) cases (in six patients, the duration of onset of symptoms was not reported; therefore, they were excluded from the final analysis).

In 18 patients (28%), the infection extended to one or more surrounding organs or tissues including diaphragm, abdominal wall, lungs, transverse colon, stomach with extension to the gastric muscularis propria, thorax, and inferior vena cava. For six other patients, it was assumed that hepatic abscess caused Thrombotic Thrombocytopenic Purpura (TTP), acute cardiac tamponade (in two cases), hepatic artery invasion with encasement of the portal vein and celiac and liver hilum adenopathies, hepatic vein and IVC thromboses, and finally right pulmonary empyema (Additional file 1: Table S1).

### Diagnosis

The diagnosis was confirmed by histopathologic examination of different specimens in 43 of 64 (67%) instances: liver puncture biopsy in 14 cases, liver lobe resection in 13 cases, laparoscopic or surgical tissue in 9 cases, and autopsy in one case. In six other patients, histopathologic examination of samples other than liver, including resected appendix, pipelle endometrial biopsy, pelvic tumor, adnexae, thoracoscopic lung biopsy, right salpingo-oophorectomy and bilateral salpingo-oophorectomy, small bowel resection, and appendectomy led to actinomycosis detection. Sulfur granules were reported from 36 (56%) patients. Although liver puncture biopsy was performed for 10 patients, it did not lead to the diagnosis of actinomycosis and in one patient, needle aspiration could not be performed, because the liver lesions suspicious for hydatid cyst were localized subcapsularly (Additional file 1: Table S1 presents all the findings from histopathological examinations).

In five (7.9%) other patients, the information obtained from culture and histopathologic examination led to the diagnosis of infection. Furthermore, culture alone diagnosed actinomycosis in seven patients (11%). Notably, culture was not used to diagnose infection in 24 (37%) patients and out of 40 patients for whom this method of diagnosis was used, only 18 (45%) cases came positive. Aspirate obtained from the hepatic abscess and tissue specimens resulted in positive culture for 12 and three patients, respectively. Blood, material spontaneously drained through a fistula between liver and skin, and pericardial fluid cultures also led to the diagnosis of actinomycosis in three other cases. The duration of positive culture was reported in only three patients, which was eight and 10 days for the aspirate obtained from the hepatic abscess of two patients and four days for pericardial fluid. Blood culture was reported in 13 instances and found positive in the detection of actinomycosis in only two (15%) cases.

**Fig. 4:**
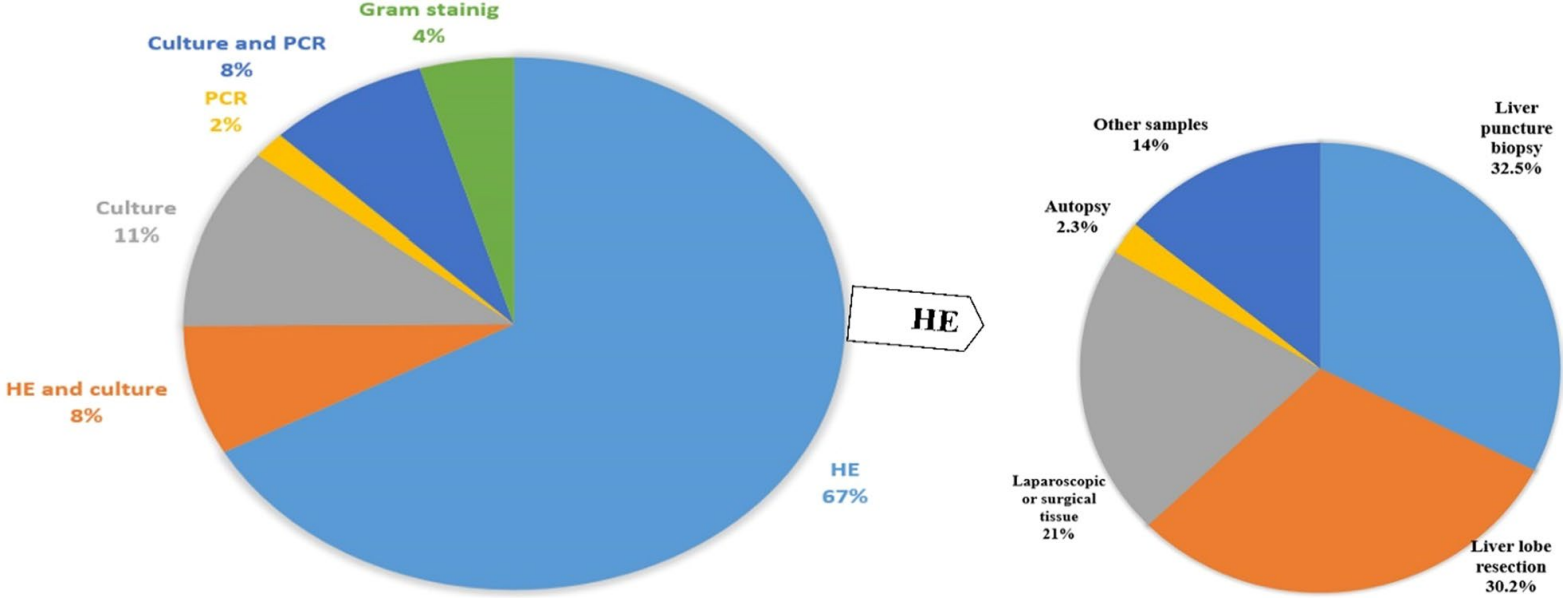
Different methods that led to the diagnosis of hepatic actinomycosis. *HE* Histopathologic examination, *PCR* polymerase chain reaction

Polymerase Chain Reaction (PCR) was used to confirm the diagnosis of infection in only one (1.6%) patient. In five (7.9%) other cases, culture system (API system in two cases and VITEK II system in two other patients) could not confirm the bacteria’s species and identification of the level of phenotypic species was impossible. Therefore, genomic identification was performed by 16S ribosomal RNA gene sequencing analysis. Finally, in three instances (4.6%) (Fig. 4), gram staining of transthoracic needle aspiration, hepatic lesion, and pleural fluid aspiration without the use of any other diagnostic method led to the identification of actinomycosis in the patients (Additional file 1: Table S1).

Medical imaging modalities of Computed Tomography (CT), Magnetic Resonance Imaging (MRI), and ultrasound did not give a definitive diagnosis for any of the patients. Among the ultrasound, MRI, and CT information for the patients, the most frequent finding was a single abscess or mass (57%). The right lobe (52%) was the most frequently affected, followed by the left lobe (26%) and both lobes (21%). The imaging characteristics of HA were mainly mass-like and nodular (70% of cases), abscess-like (67%), low density (50%) and solid tumor-like (32%). In addition, a few (26%) hypodense cystic cases were observed. The available information on medical imaging modalities is shown in Additional file 1: Table S2.

Information from the patients’ clinical signs, imaging, and liver puncture biopsy led to the suspicion of liver malignant tumor or liver metastasis, hepatic inflammatory pseudotumor, and pyogenic liver abscesses in 32 (50%), seven (11%) and 20 (31%) cases, respectively (Fig. 3). All the findings that gave the diagnosis of HA in patients are fully provided in Tables S1 and S2.

### Treatment

From the data available in the literature, in a total of 64 cases, surgery or puncture drainage + anti-infection, liver lobe resection + anti-infection, anti-infection alone, and liver lobe resection alone were used for 30 (46%), 14 (21%), 18 (28%), and one patient, respectively. One of the patients died before starting the treatment and in another case, no information was reported about the type of drug the patient was taking. Therefore, they were excluded from the final analysis. Antibiotics including penicillin (32 patients, 51%), Amoxicillin (17 patients, 27%), Doxycycline and ampicillin (six patients, 9%) were the most frequently used drugs for the treatment of HA in patients (Fig. 3). Duration of treatment was state in 45 cases and the mean duration of treatment was 5.5 months (mean ± SD = 5.5 ± 3.8 months ranging from one week to 24 months). As mentioned, with regard to the clinical and laboratory symptoms, pyogenic liver abscesses were suspected in a number of patients. For this reason, the use of empirical therapies to cover a wide range of bacteria was prescribed for them. However, in almost all of the cases, no improvement was achieved. Additional file 1: Table S3 covers these empirical treatments and their implications.

## Discussion

HA is a very rare abdominal actinomycosis type and generally occurs after abdominal infection [36, 38]. Our results showed any age group could be infected, although patients over the age of 55 seemed to be more prone to this infection. Also, the rate of infection was higher in men between 50 and 70 years as the most susceptible group. The reason for the male predominance was uncertain, but all forms of actinomycosis were recognized in observations [61]. Previous studies also reported that HA was usually seen in males, but the most common age range for the infection in these studies was 30–50 years [47, 71]. These studies were conducted in more distant years than the present research (1990–2005). Therefore, it seems that in recent years, the average age of men for HA has increased. On the other hand, females have been more often affected probably due to a long-term use of the IUD and endometrial colonization with *A. israelii* [51, 72]. Our analysis showed that although the diagnosis was often made late and as a result, a delay occurred in initiating the therapy, the outcome was totally acceptable and the mortality rate was 1.5%. (1/64). In a recent study that was conducted with HA cases from 1966 to 2002, the overall mortality rate was 8.8% (5/57) [61]. Therefore, it seems that due to the good response of HA to treatment, there has been no significant change in the mortality rate in recent years.

Our finding showed that most of the patients experienced complications, the most frequent cases of which were a previous history of abdominal surgery, pelvic colonization due to the use of an IUD, and oral diseases or dental surgery. HA often occurs through the portal vein after the mechanical disruption of the intestinal normal physical barriers in case of surgery intervention and mucosal trauma or via the hepatic artery during hematogenous spread from an oral source [27, 73, 74]. Thus, patients with poor oral hygiene, history of IUD, or intra-abdominal surgery should be evaluated further for HA. Although for preventing HA, we do not have specific measures yet, keeping good personal dental hygiene and, specifically, removing dental plaque may be effective in reducing the density. However, effectiveness of such measures in reducing the probability of incidence of colonization and low-grade periodontal infection with *Actinomyces* species is not proved [75]. In 13 cases, no disorders were reported that could predispose a patient to HA. Therefore, the liver can be involved without disrupting the tissue barriers [71]. A recent study reported that the infiltrative nature and rapid progression of actinomycosis along with its potential for invading the normal anatomic barriers was probably due to the proteolytic enzymes of *Actinomyces* species [50]. These microorganisms, by using proteolytic enzymes, can propagate from the liver towards diaphragm and lungs or penetrate the pleural space through lymph vessels or pleuro-peritoneal communications on the diaphragm [42, 76]. Thus, *Actinomyces* species secrete proteolytic enzymes, penetrate even normal anatomic barriers, and infiltrate the diaphragm; they are complicated by pleural and lung lesions, make cutaneous fistula, and extend to the abdominal and pelvic organs [31].

The clinical signs of HA are usually nonspecific and similar to other infections. However, abdominal pain, fever, and weight loss are the most reported signs among the patients. Anemia and leukocytosis are also other symptoms reported in more than half of the patients. Unlike pyogenic abscess in which 63% of patients have elevated AST levels and 70% of patients have elevated ALT levels, based on the findings of this study, less than one-third of the patients have elevated AST and ALT levels with HA [47, 77]. Increased levels of CA19-9 and CA-125 were reported in five cases. Our finding showed that in patients with HA, tumor marker levels were lower (50–278 U/ml) than those with malignant conditions and at levels greater than 1000 U/ml, the marker’s positive predictive value approached 100% [30]. This might be due to the enhanced production of CA 19–9 from biliary epithelial cells, which is prevalent in the course of benign diseases of the biliary tract, and/or the reduction in hepatobiliary clearance because cholestasis may have led to the increased levels of CA 19–9 in the blood [30]. However, CA19-9 has inadequate sensitivity and specificity to be used as a marker for differentiating HA from liver tumors and appropriate consideration of this marker might turn out useful in the difficult diagnosis. A study has reported that high mobility group box chromosomal protein 1 (HMGB1) may have a potent biological effect on the pathogenesis of HA as a novel cytokine and may be a useful marker in the differential diagnosis of HA [22]. Thus, this marker deserves higher attention in future studies. Clinical manifestation and laboratory tests are non-specific in patients with HA and correct diagnosis of infection is the most difficult step in controlling this infection.

In none of the cases, the imaging modalities result in a definitive diagnosis of infection in the patients. As mentioned, on CT, HA is mainly seen as a single or multiple low-density shadows, which may be accompanied by enhancement. The border is often unclear in this type of images. Thus, it is very difficult to distinguish HA from ordinary bacterial abscesses, inflammatory masses, solitary metastatic lesions, and primary liver masses on CT or MRI [32, 63]. In this context, in eight cases (12%), misdiagnosis based on imaging information led to surgery in patients and perhaps if the HA had been correctly diagnosed in these patients, extensive surgery would not have been necessary at all [12, 20–23, 50, 53, 63]. In another patient, lesions in liver were not detected by the CT scan either, because they were only approximately 0.5 cm in size, which was beyond the limitations of CT cutting by 1 cm [1]. On the other hand, two studies reported that the use of Contrast Enhanced Ultrasonography (CEUS) might help demonstrate the inflammatory substrate of the lesion and could be very useful for visualizing the lesions and evaluating central liquefaction during the arterial phase as well as the status of the disease. Thus, this technique should be more investigated in future patients [32, 41].

Due to the limitations that exist for imaging modalities, the final diagnosis is made on patohystology, which is the gold standard to confirm or exclude the diagnosis of HA [42]. Histopathologic examination of different samples confirmed the diagnosis of HA in 67% of the patients. On the other hand, histopathologic examination of samples from liver puncture biopsy and liver lobe resection did not help diagnose actinomycosis in 10 and one patient, respectively. Recent studies have reported that, without absolute bacterial identification, the most helpful diagnostic finding is the detection of sulfur granules, also confirmed in the present study with 56% of the patients [38, 78]. However, when only small quantities of tissues are available, sulfur granules can be easily missing and only inflammation or fibrosis may be identified [8]. Therefore, per-cutaneous biopsy diagnosis is not always fruitful and the organism cannot be seen, because the typical sulfur granules are present in the part of the lesion that the biopsy does not reach. Hence, it is recommended that an exploratory laparotomy be adopted for exact diagnosis and treatment [21]. On Hematoxylin and Eosin (H&E) stains, the bacteria are clumped by the radiating fringe of club-like organisms in the sulfur granules and surrounded by neutrophils and lymphocytes. Grocott-Gomori methenamine silver staining is used to identify branching organisms that characterize the presence of actinomycosis. Multinucleated giant cells or granulomas may be seen in a number of cases. However, these morphological results are not specific to actinomycosis, since other microorganisms like *Nocardia* species and certain fungal or parasitic infections can lead to similar morphological outcomes [1, 34]. Therefore, histopathologic examination of different samples also has limitations for diagnosis and, for some patients, we need to use other ways such as culture and molecular methods for definitive diagnosis of infection.

Our analysis showed that culture was not used to diagnose HA in 37% of the patients and positive results were reported in only 18 patients. The failure rate of culture is quite high often due to growth suppression caused by prior antibiotic therapy, improper specimen gathering and transportation techniques, lack of the proper media, and inadequate culture conditions [8, 70]. On the other hand, while a recent study has reported that positive peripheral blood culture is one of the diagnostic methods for actinomycosis in patients, our results indicated that only two patients had a positive blood culture [37, 61]. Thus, it is difficult to culture *Actinomyces* while, isolating bacteria from clinical specimens may be necessary to separate nocardiosis or botryomycosis from actinomycosis, diseases that are often morphologically difficult to distinguish. Therefore, direct inoculation of aspirated pus or liver biopsy material into both aerobic and anaerobic blood culture samples may elevate culture sensitivity [79].

The use of molecular methods can be helpful in diagnosing HA, although for only six cases (9%) PCR was used according to the findings of the present study. In one patient, ultrasound guided liver biopsy and diagnostic laparoscopy with excisional biopsy of a lesion did not make an HA diagnosis. Due to the positive Quantiferon-TB test, the liver lesions were thought secondary to disseminated reactivated tuberculosis and the patient underwent RIPE therapy. In this patient, who had a very complex condition, the use of PCR resulted in the correct diagnosis of *Actinomyces* DNA and hence, the use of appropriate therapies [70]. Accordingly, it can be concluded that each method of diagnosing HA has its limitations and many patients undergo hepatectomy and other extensive surgery due to the difficulty in detecting *Actinomyces* and distinguishing between *Actinomyces* abscesses and malignant tumors. Recently, new molecular genetic methods such as PCR for 16S rRNA sequencing and fluorescence in situ hybridization have been developed to achieve faster and more accurate identification in reference or research laboratories [32, 80]. Such methods are highly recommended when there are many challenges to diagnosing HA in the patient.

With correct diagnosis of this infection in patients, the response for treatment was usually good and our finding showed only one death from HA [56]. On the other hand, HA could easily extend transdiaphragmatically to the pericardium and provoke serious illness. Thus, early definitive diagnosis and appropriate antibiotics therapy are critically important [9]. In this context, in one patient, the failure in the first therapy led to transdiaphragmatic spread of HA and its extension into the pericardium caused acute cardiac tamponade [9]. It is noteworthy that *Actinomyces* spp. are usually highly susceptible to beta-lactams, especially penicillin G or Amoxicillin. Amoxicillin offers high potency in diffusing into inflamed liver tissue and is thus the preferable compound for treating HA [25].

Surgery is another treatment strategy used for patients with HA. There have been many controversies in previous studies about how to use surgery. Our findings showed that 28% of the patients were treated with anti-infection alone and only one case was treated with liver lobe resection alone. One study reported that the outcome of hepatectomy was excellent since no recurrence was observed [27]. Another case also reported that initial treatment with benzyl penicillin and imipenem/cilastatin was ineffective and an exploratory laparotomy and right lobectomy of the liver were performed [49]. Hence, surgery needs to be adopted for the cases in which percutaneous drainage is not possible, for larger lesions in which there is a greater amount of necrotic tissue, and when symptoms are not improved or are aggravated after treatment [28, 36]. On the other hand, as mentioned, in some cases, extended resection was unnecessarily performed. Hence, using preoperative empirical antibiotics and excluding malignant tumors during surgery via frozen biopsy are recommended. Applying this strategy can ensure a reduction in the extent of surgery and postoperative complications in patients with actinomycosis indistinguishable from malignant tumor before surgery [38]. Finally, it should be noted that because of the tendency of the disease to recur, the antibiotic treatment should incorporate high doses and have a prolonged duration. A long-term follow-up is also required to monitor the treatment response and detect any recurrence earlier. The duration of treatment should be individualized based on a number of factors including disease location, severity, and changes in follow-up imaging.

## Limitation

The present study included only PubMed/Medline studies available in English language, which contained an abstract, hence reducing the number of relevant publications. It was not possible to discuss the bias, risks, or individual limitations of the studies, since they were not reported.

## Conclusion

HA is a difficult disease to diagnose due to its rarity, nonspecific symptoms, failure of identifying the microorganism, and imitation of more common conditions. In many patients, this infection is confused with other diseases, which may lead to extensive surgery. Therefore, it is recommended that HA be diagnosed correctly before deciding on surgery by using a variety of diagnostic methods considering the fact that, in many patients, infection can be controlled using only antibiotics. Finally, due to the tendency of the disease to recur, the antibiotic treatment must incorporate high doses and have a prolonged duration. A long-term follow-up is also necessary to monitor the treatment response and detect any recurrence early. The duration of treatment should be individualized based on disease location, severity, and changes in follow-up imaging.

## Supplementary Information

**Additional file 1: Table S1**. Various findings that led to the diagnosis of hepatic actinomycosis in different patients. **Tables S2**. Radiological and anatomic characteristics of hepatic actinomycosis. **Tables S3**. Prophylaxis and Empirical antibiotics therapy in patients with hepatic actinomycosis. **Tables S4**. Microbiological features of hepatic actinomycosis.

## Data Availability

The authors confirm that the data supporting the findings of this study is available within the article and its supplementary materials.

## References

[1] Wang H-K, Sheng W-H, Hung C-C, Chen Y-C, Liew P-L, Hsiao C-H, Chang S-C (2012). Hepatosplenic actinomycosis in an immunocompetent patient. J Formos Med Assoc.

[2] Joshi V, Koulaouzidis A, McGoldrick S, Tighe M, Tan C (2010). Actinomycotic liver abscess: a rare complication of colonic diverticular disease. Ann Hepatol.

[3] Kanellopoulou T, Alexopoulou A, Tanouli MI, Tiniakos D, Giannopoulos D, Koskinas J, Archimandritis AJ (2010). Primary hepatic actinomycosis. Am J Med Sci.

[4] Ávila F, Santos V, Massinha P, Pereira JR, Quintanilha R, Figueiredo A, Lázaro A, Carrelho S, Coelho JS, Barroso E (2015). Hepatic actinomycosis. GE Port J Gastroenterol.

[5] Wong JJ, Kinney TB, Miller FJ, Rivera-Sanfeliz G (2006). Hepatic actinomycotic abscesses: diagnosis and management. AJR Am J Roentgenol.

[6] Smith AJ, Hall V, Thakker B, Gemmell CG (2005). Antimicrobial susceptibility testing of Actinomyces species with 12 antimicrobial agents. J Antimicrob Chemother.

[7] Valour F, Senechal A, Dupieux C, Karsenty J, Lustig S, Breton P, Gleizal A, Boussel L, Laurent F, Braun E (2014). Actinomycosis: etiology, clinical features, diagnosis, treatment, and management. Infect Drug Resist.

[8] Ha YJ, An JH, Shim JH, Yu ES, Kim JJ, Ha TY, Lee HC (2015). A case of primary hepatic actinomycosis: an enigmatic inflammatory lesion of the liver. Clin Mol Hepatol.

[9] Sakaguchi Y, Isowa N, Nakazaki H, Takeda K, Tokuyasu H, Saitoh Y, Soeda T, Ohe T, Tokuyasu Y, Miura H (2012). Acute cardiac tamponade caused by the extension of multiple hepatic actinomycotic abscesses. Intern Med.

[10] Lall T, Shehab TM, Valenstein P (2010). Isolated hepatic actinomycosis: a case report. J Med Case Reports.

[11] Aslan A, Ayaz E, Inan I, Acar M (2018). Isolated hepatic actinomycosis mimicking hepatocellular carcinoma: Case report and review. AIDM.

[12] Yang X-X, Lin J-M, Xu K-J, Wang S-Q, Luo T-T, Geng X-X, Huang R-G, Jiang N (2014). Hepatic actinomycosis: report of one case and analysis of 32 previously reported cases. World J Gastroenterol.

[13] Wong VK, Turmezei T, Weston V (2011). Actinomycosis. BMJ.

[14] Hickey AJ, Gounder L (2015). Moosa M-YS, Drain PK: a systematic review of hepatic tuberculosis with considerations in human immunodeficiency virus co-infection. BMC Infect Dis.

[15] Chegini Z, Didehdar M, Khoshbayan A, Rajaeih S, Salehi M, Shariati A (2020). Epidemiology, clinical features, diagnosis, and treatment of cerebral mucormycosis in diabetic patients: a systematic review of case reports and case series. Mycoses.

[16] Moola S, Munn Z, Tufanaru C, Aromataris E, Sears K, Sfetcu R, Currie M, Qureshi R, Mattis P, Lisy K. Chapter 7: systematic reviews of etiology and risk. *Joanna Briggs Institute Reviewer's Manual The Joanna Briggs Institute* 2017:2019–2005.

[17] Alawainati M, Al-Khawaja S, Shawqi Z, Alshaikh S (2020). Disseminated actinomycosis a rare cause of abdominal pain: a case report. Oman Med J.

[18] Tambay R, Côté J, Bourgault A-M, Villeneuve J-P (2001). An unusual case of hepatic abscess. Can J Gastroenterol.

[19] Lawson E (2005). Systemic actinomycosis mimicking pelvic malignancy with pulmonary metastases. Can Respir J.

[20] Lai AT, Lam CM, Ng KK, Yeung C, Ho WL, Poon LT, Ng IO (2004). Hepatic actinomycosis presenting as a liver tumour: case report and literature review. Asian J Surg.

[21] Yu C-Y, Chang W-C, Gao H-W, Chao T-Y, Huang G-S, Hsieh C-B (2010). Metastatic hepatic actinomycosis. Am J Med.

[22] Wu C-X, Guo H, Gong J-P, Liu Q, Sun H (2013). The role of high mobility group box chromosomal protein 1 expression in the differential diagnosis of hepatic actinomycosis: a case report. J Med Case Rep.

[23] Zeng QQ, Zheng XW, Wang QJ, Yu ZP, Zhang QY (2018). Primary hepatic actinomycosis mimicking liver tumour. ANZ J Surg.

[24] Hernigou J, Dugué L, Maftouh A, Balian C, Charlier A (2013). Appendiceal actinomycosis complicated by multiple hepatic abscesses. J Visc Surg.

[25] Lange C, Hofmann W, Kriener S, Jacobi V, Welsch C, Just-Nuebling G, Zeuzem S (2009). Primary actinomycosis of the liver mimicking malignancy. Z Gastroenterol.

[26] Harsch I, Benninger J, Niedobitek G, Schindler G, Schneider H, Hahn E, Nusko G (2001). Abdominal actinomycosis: complication of endoscopic stenting in chronic pancreatitis?. Endoscopy.

[27] Felekouras E, Menenakos C, Griniatsos J, Deladetsima I, Kalaxanis N, Nikiteas N, Papalambros E, Kordossis T, Bastounis E (2004). Liver resection in cases of isolated hepatic actinomycosis: case report and review of the literature. Scand J Infect Dis.

[28] Kanellopoulou T, Alexopoulou A, Tiniakos D, Koskinas J, Archimandritis AJ (2010). Primary hepatic actinomycosis mimicking metastatic liver tumor. J Clin Gastroenterol.

[29] Lakshmana Kumar Y, Javherani R, Malini A, Prasad S (2005). Primary hepatic actinomycosis. Trans R Soc Trop Med Hyg.

[30] Soardo G, Basan L, Intini S, Avellini C, Sechi LA (2005). Elevated serum CA 19–9 in hepatic actinomycosis. Scand J Gastroenterol.

[31] Uehara Y, Takahashi T, Yagoshi M, Shimoguchi K, Yanai M, Kumasaka K, Kikuchi K (2010). Liver abscess of *Actinomyces israelii* in a hemodialysis patient: case report and review of the literature. Intern Med.

[32] Oe S, Shibata M, Hiura M, Mitsuoka H, Matsuhashi T, Narita R, Abe S, Tabaru A, Hayashida K, Taniguchi H (2014). Refractory primary hepatic actinomycosis with direct infiltration to the diaphragm and thorax: the usefulness of contrast-enhanced ultrasonography. Intern Med.

[33] Ishiguro T, Takayanagi T, Ikarashi H (2016). Multiple metastatic liver abscesses and intravenous thrombosis due to pelvic actinomycosis. Eur J Obstet Gynecol Reprod Biol.

[34] Kim HS, Park NH, Park KA, Kang SB (2007). A case of pelvic actinomycosis with hepatic actinomycotic pseudotumor. Gynecol Obstet Invest.

[35] Min KW, Paik SS, Han H, Jang KS (2012). Hepatobiliary and pancreatic: hepatic actinomycosis. J Gastroenterol Hepatol.

[36] Kim YS, Lee BY, Jung MH (2012). Metastatic hepatic actinomycosis masquerading as distant metastases of ovarian cancer. J Obstet Gynaecol Res.

[37] Seo JY, Yeom J-S, Ko KS (2012). Actinomyces cardiffensis septicemia: a case report. Diagn Microbiol Infect Dis.

[38] Yang SS, Im YC (2018). Severe abdominopelvic actinomycosis with colon perforation and hepatic involvement mimicking advanced sigmoid colon cancer with hepatic metastasis: a case study. BMC Surg.

[39] Lee JH, Kim HS, Kim JS, Lee DK, Lim JH (2018). Hepatic actinomycosis with immunoglobulin G4-related liver disease: a case report. Medicine.

[40] Petrache D, Popescu G-A (2013). Successful switch to oral therapy with doxycycline in the case of an actinomycotic hepatic abscess. J Infect Dev Ctries.

[41] Badea R, Chiorean L, Matei D, Seicean A, Andreica V, Botan E (2013). Accidentally ingested foreign body associated with liver actinomycosis: the diagnostic value of imaging. J Gastrointestin Liver Dis.

[42] Culafić DM, Lekić NS, Kerkez MD, Mijac DD (2009). Liver actinomycosis mimicking liver tumour. Vojnosanit Pregl.

[43] Basaric D, Lekic N, Djordjevic V, Ceranic M, Barac A, Stevanovic G, Milosevic I (2018). Actinomycotic hepatic abscess in woman with longstanding intrauterine contraceptive device. J Infect Dev Ctries.

[44] Llenas-García J, Lalueza-Blanco A, Fernández-Ruiz M, Villar-Silva J, Ochoa M, Lozano F, Lizasoain M, Aguado J (2012). Primary hepatic actinomycosis presenting as purulent pericarditis with cardiac tamponade. Infection.

[45] Correa Bonito A, Mora-Guzmán I, García-Sanz I, di Martino M, Martín-Pérez E (2017). Liver abscess after endoscopic retrograde cholangiopancreatography with presence of *Actinomyces naeslundii*. Cir Esp.

[46] Lin T-P, Fu L-S, Peng H-C, Lee T, Chen J-T, Chi C-S (2001). Intra-abdominal actinomycosis with hepatic pseudotumor and xanthogranulomatous pyelonephritis in a 6-y-old boy. Scand J Infect Dis.

[47] Chen LW, Chang LC, Shie SS, Chien RN (2006). Solitary actinomycotic abscesses of liver: report of two cases. Int J Clin Pract.

[48] Chao C-T, Liao C-H, Lai C-C, Hsueh P-R (2011). Liver abscess due to *Actinomyces odontolyticus* in an immunocompetent patient. Infection.

[49] Li P-F, Huang D-W, Peng C-K (2014). Computed tomography of an *Actinomyces israelii* liver abscess. QJM.

[50] Cetinkaya Z, Kocakoc E, Coskun S, Ozercan IH (2010). Primary hepatic actinomycosis. Med Princ Pract.

[51] Coban A, Yetkin G, Kebudi A (2003). Abdominal actinomycosis: a case report. Acta Chir Belg.

[52] Buyukavci M, Caner I, Eren S, Aktas O, Akdag R (2004). A childhood case of primary hepatic actinomycosis presenting with cutaneous fistula. Scand J Infect Dis.

[53] Gonenc K, Atahan C, Haluk E, Betul T, Aydin A, Semra C (2006). A case of isolated hepatic actinomycosis causing right pulmonary empyema. Chin Med J.

[54] Guven A, Kesik V, Deveci MS, Ugurel MS, Ozturk H, Koseoglu V (2008). Post varicella hepatic actinomycosis in a 5-year-old girl mimicking acute abdomen. Eur J Pediatr.

[55] Tiftikci A, Vardareli EN, Kaban K, Peker O, Akansel S, Tozun N (2008). Actinomycotic hepatic abscess. Hepatol Int.

[56] Powell G, Mangalika M. Thoraco-pulmonary and hepatic actinomycosis: an autopsy report. Case Rep. 2011;2011:bcr0920114831. 10.1136/bcr.09.2011.4831 PMC3214224 22674116

[57] O’Kelly K, Abu J, Hammond R, Jensen M, O’Connor RA, Soomro I (2012). Pelvic actinomycosis with secondary liver abscess, an unusual presentation. Eur J Obstet Gynecol Reprod Biol.

[58] Mukundan G, Fishman EK (2000). Pulmonary and hepatic actinomycosis: atypical radiologic findings of an uncommon infection. Clin Imaging.

[59] Hilfiker ML (2001). Disseminated actinomycosis presenting as a renal tumor with metastases. J Pediatr Surg.

[60] Riegert-Johnson DL, Sandhu N, Rajkumar SV, Patel R (2002). Thrombotic thrombocytopenic purpura associated with a hepatic abscess due to *Actinomyces turicensis*. Clin Infect Dis.

[61] Sharma M, Briski LE, Khatib R (2002). Hepatic actinomycosis: an overview of salient features and outcome of therapy. Scand J Infect Dis.

[62] Islam T, Athar MN, Athar MK, Usman MHU, Misbah B (2005). Hepatic actinomycosis with infiltration of the diaphragm and right lung: a case report. Can Respir J.

[63] Wayne MG, Narang R, Chauhdry A, Steele J (2011). Hepatic actinomycosis mimicking an isolated tumor recurrence. World J Surg Oncol.

[64] Jaqua NT, Smith AJ, Shin TT, Jahanmir J. Actinomyces naeslundii and Eikenella corrodens as rare causes of liver abscesses. Case Rep. 2013;2013:bcr2013009613. 10.1136/bcr-2013-009613 PMC3736225 23867879

[65] Sahay S, McKelvy BJ (2017). Actinomycosis presenting as recurrent hepatic abscess. Am J Med.

[66] Xing J, Rodriguez EF, Monaco SE, Pantanowitz L (2016). Cytopathology of hepatobiliary-related actinomycosis. Acta Cytol.

[67] Kothadia JP, Samant H, Olivera-Martinez M (2018). Actinomycotic hepatic abscess mimicking liver tumor. Clin Gastroenterol Hepatol.

[68] Ridha A, Oguejiofor N, Al-Abayechi S, Njoku E. Intra-abdominal actinomycosis mimicking malignant abdominal disease. Case Rep Infect Dis. 2017;2017:1972023. 10.1155/2017/1972023 PMC5337322 28299215

[69] Grossen A, Magguilli M, Thai TC, Salem G. Hepatic actinomycosis in a patient with retained common bile duct stent. ACG Case Rep J. 2019;6(9):e00219. 10.14309/crj.0000000000000219 PMC6831134 31750385

[70] Murphy P, Mar WA, Allison D, Cornejo GA, Setty S, Giulianotti PC (2020). Hepatic actinomycosis–A potential mimicker of malignancy. Radiol Case Rep.

[71] Miyamoto MI, Fang FC (1993). Pyogenic liver abscess involving Actinomyces: case report and review. Clin Infect Dis.

[72] Meyer P, Nwariaku O, McCelland R, Gibbons D, Leach F, Sagalowsky AI, Simmang C, Jeyarajah D (2000). Rare presentation of actinomycosis as an abdominal mass. Dis Colon Rectum.

[73] Kasano Y, Tanimura H, Yamaue H, Hayashido M, Umano Y (1996). Hepatic actinomycosis infiltrating the diaphragm and right lung. Am J Gastroenterol.

[74] Cheng YF, Hung CF, Liu YH, Ng KK, Tsai CC (1989). Hepatic actinomycosis with portal vein occlusion. Gastrointest Radiol.

[75] Smego RA, Foglia G (1998). Actinomycosis. Clin Infect Dis.

[76] Ubeda B, Vilana R, Bianchi L, Pujol T (1995). Primary hepatic actinomycosis: association with portal vein thrombosis. AJR Am J Roentgenol.

[77] Sugano S, Matuda T, Suzuki T, Makino H, Iinuma M, Ishii K, Ohe K, Mogami K (1997). Hepatic actinomycosis: case report and review of the literature in Japan. J Gastroenterol.

[78] Lee JD, Kim PG, Jo H, Park DH, Seo E (1993). A case of primary hepatic actinomycosis. J Korean Med Sci.

[79] Runyon BA, Canawati HN, Akriviadis EA (1988). Optimization of ascitic fluid culture technique. Gastroenterology.

[80] Hansen J, Fjeldsøe-Nielsen H, Sulim S, Kemp M, Christensen J (2009). Actinomyces species: a Danish survey on human infections and microbiological characteristics. Open Microbiol J.

