# Supplementary material for: A systematic review of case reports of hepatic actinomycosis
Source: Orphanet J Rare Dis. 2021 Apr 30;16:192. doi: 10.1186/s13023-021-01821-5 (PMC8086304; doi:10.1186/s13023-021-01821-5)
Supplement: Supplementary file 1 — Additional file 1: Table S1. Various findings that led to the diagnosis of hepatic actinomycosis in different patients. Tables S2. Radiological and anatomic characteristics of hepatic actinomycosis. Tables S3. Prophylaxis and Empirical antibiotics therapy in patients with hepatic actinomycosis. Tables S4. Microbiological features of hepatic actinomycosis. [file 13023_2021_1821_MOESM1_ESM.docx]

**Table S1:** Various findings that led to the diagnosis of hepatic actinomycosis in different patients.

| **Country, year of publication and references** | **Liver puncture fluid or drainage** | **Liver puncture biopsy** | **Liver lobe resection** | **Laparoscopic or surgical tissue** | | **Other** | **Autopsy** | **Culture** | **confirmation** |
| --- | --- | --- | --- | --- | --- | --- | --- | --- | --- |
| **Bahrain, 2020**  **[17]** | NA | NA | NA | | Extensive inflammation with lymphoplasmacytic infiltrates, macrophages, abscesses, and Actinomyces | NA | NA | NA | HE |
| **Canada, 2001**  **[18]** | NA | NA | NA | | NA | A thoracoscopic lung biopsy showed abscesses containing aggregates of Actinomyces species | NA | Negative | HE |
| **Canada, 2005**  **[19]** | NA | Sulphur granules of actinomycosis infection (center) in a neutrophil-rich inflammatory background | NA | | NA | NA | NA | Positive of hepatic absecces | HE and culture |
| **China, 2004**  **[20]** | NA | NA | multiple collections of heavy acute and chronic inflammatory infiltrates with florid fibrosis. Notably, a few clusters of filamentous microorganisms | | NA | NA | NA | Negative | HE |
| **China, 2011**  **[21]** | NA | NA | Presence of sulfur granules by hematoxylin–eosin stain, characteristic of an Actinomyces israelii infection | | NA | NA | NA | NA | HE |
| **China, 2013**  **[22]** | NA | NA | Sulfur granules surrounded by neutrophils, foaming histiocytes, lymphocytes, and plasma cells | | NA | NA | NA | NA | HE |
| **China, 2014**  **[12]** | NA | NA | Multifocal actinomycetes in the hepatic parenchyma, accompanied by chronic suppurative inflammation, and a focal abscess | | NA | NA | NA | NA | HE |
| **China, 2016**  **[23]** | NA | NA | Multiple aggregates of acute and chronic inflammatory infiltrates with florid fibrosis and several clusters of filamentous microorganisms consistent with actinomyces Sulphur granules | | NA | NA | NA | NA | HE |
| **France, 2013**  **[24]** | NA | NA | NA | | NA | The pathology results of the resected appendix demonstrated an actinomycotic infection. | NA | NA | HE |
| **Germany, 2009**  **[25]** | NA | Necrotic lesions with activated macrophages and granulocytes, sulfur granules and Gram-positive filamentous bacteria forming radiating aggregates | NA | | NA | NA | NA | Negative | HE |
| **Germany,2001**  **[26]** | NA | Liver parenchyma and scar tissue had distinct florid inflammation. | NA | | NA | NA | NA | Negative | HE |
| **Greece, 2004**  **[27]** | NA | Not diagnostic | The detection of few grains consistent with actinomycetes allowed the diagnosis of actinomycosis of the liver | | NA | NA | NA | Negative | He |
| **Greece, 2010**  **[28]** | NA | Prominent lobular inflammation with large areas of hepatocyte loss, necrosis, and bacterial colonies of Actinomyces surrounded by polymorphs | NA | | NA | NA | NA | NA | HE |
| **India, 2005**  **[29]** | Gram stain of the pus showed numerous pus cells and thin filamentous, non-acid fast, branching Gram-positive organisms, breaking into coccoid and bacillary fragments at places. | NA | NA | | NA | NA | NA | Positive of liver absecces after 8 days | culture |
| **Italy, 2005**  **[30]** | NA | Not diagnostic | Sulphur granules were identified microscopically consistent with the presence of filamentous organisms that stained positively with Gram, PAS and GMS stain but were acid-fast negative, as Actinomyces spp | | NA | NA | NA | Positive from liver mass pus | HE |
| **Japan, 2011**  **[31]** | culture of hepatic mass aspirate | NA | NA | | NA | NA | NA | Positive of liver drainage after 10 days | culture and PCR |
| **Japan, 2012**  **[9]** | NA | This pathological finding is compatible with actinomyces druse and sulfur granule | NA | | NA | NA | NA | Negative | HE |
| **Japan, 2014**  **[32]** | NA | An abscess-forming dense acute inflammatory infiltrate and colonies of Actinomyces with tiny radiate granules and peripheral eosinophilic clubs | NA | | NA | NA | NA | NA | HE |
| **Japan, 2016**  **[33]** | not detected infection | NA | NA | | NA | Pelvic tumor showed acute oophorosalpingitis with several abscesses due to actinomycosis |  | Negative | HE |
| **Kore, 2006**  **[34]** | NA | Not diagnostic | NA | | NA | Colonies of Actinomyces species and sulfur granules were detected with surrounding inflammatory infiltration by microscopic examination of bilateral adnexae | NA | Negative | HE |
| **Korea, 2012**  **[35]** | blue colonies of actinomycosis with “bales of wool” appearance were seen on a background of mixed inflammatory cells | Many irregularly lobulated or scalloped basophilic granules termed “Sulphur granules” that are characteristic of Actinomyces. | NA | | NA | NA | NA | NA | HE |
| **Korea, 2012**  **[36]** | NA | NA | NA | | NA | A right salpingo-oophorectomy actinomycotic colony demonstrating the characteristic sulfur granules | NA | NA | HE |
| **Korea, 2012**  **[37]** |  | NA | NA | | NA | NA | NA | Positive from blood | culture and PCR |
| **Korea, 2013**  **[8]** | NA | Not diagnostic | NA | | Exploratory laparotomy and get biopsy chronic inflammation with abscess and sulfur granule on hematoxylin and eosin stain | NA | NA | Negative | HE after three time |
| **Korea, 2018**  **[38]** | NA | NA | NA | | NA | A definitive diagnosis of colonic actinomycosis, which showed the granular colonies of bacteria, commonly termed sulfur granule, with aggregates of filamentous bacteria and neutrophils (bilateral salpingo-oophorectomy, small bowel resection and appendectomy) | NA | Negative | HE |
| **Korea, 2018**  **[39]** | NA | Not diagnostic | Dense lymphoplasmacytic infiltration with fibrosis and abscess formation, and Actinomycotic colonies in hepatic and lung parenchyma | | NA | NA | NA | NA | HE |
| **Portugal, 2014**  **[4]** | NA | Not diagnostic | Abscesses due to Actinomyces | | NA | NA | NA | NA | HE |
| **Romania, 2012**  **[40]** | NA | Acute and chronic inflammation, granulation tissue, and Gram-positive branching filamentous rods consistent with an Actinomyces species |  | | NA | NA | NA | NA | HE |
| **Romania, 2013**  **[41]** | NA | Actinomyces, surrounded by inflammatory infiltrate, mostly composed of white cells | NA | | NA | NA | NA | NA | HE |
| **Serbia, 2009**  **[42]** | Yielded nothing | NA | Area of abscess with Sulphur granules showing actinomycotic aggregates | | NA | NA | NA | NA | HE |
| **Serbia, 2018**  **[43]** | NA | NA | NA | | Abscesses due to Actinomyces with typical sulfur granules, many histiocytes and rare multinuclear giant cells | NA | NA | NA | HE |
| **Spain, 2011**  **[44]** | NA | NA | NA | | NA | culture of pericardial fluid yielded Actinomyces species (identified by the API 20A System, bioMerieux Vitek, Hazelwood, MO) | NA | Positive from pericardial fluid after 4 days | Culture and PCR |
| **Spain, 2017**  **[45]** | culture of hepatic mass aspirate |  | NA | | NA | NA | NA | Positive from liver drainage | Culture |
| **Taiwan, 2001**  **[46]** | NA | NA | NA | | Sulfur granules with branched filament surrounded by multiple foamy cells and plasma cell infiltration in the renal tissue. | NA | NA | NA | HE |
| **Taiwan, 2005**  **[47]** | Yielded nothing | Inflammatory cells, reactive fibrosis tissue and sulfur granule in one portal area were microscopically identified | NA | | NA | NA | NA | Negative | HE |
| **Taiwan, 2005**  **[47]** | NA | NA | An abscess with a thick fibrous wall containing multiple sulfur granules compatible with actinomycotic abscess | | NA | NA | NA | Negative | HE |
| **Taiwan, 2010**  **[48]** | Large amounts of coryneform Gram-positive bacilli and culture | NA | NA | | NA | NA | NA | Positive from liver absecces drainage | culture and PCR |
| **Taiwan, 2013**  **[49]**  **55** | NA | NA | *Actinomyces israelii* liver abscess | | NA | NA | NA | NA | HE |
| **Taiwan,2009**  **[1]** | NA | NA | NA | | Acute purulent and chronic inflammatory cell infiltration admixed with necrotic substance. Some microorganisms with sulfur granules | NA | NA | Negative | HE |
| **Turkey 2010**  **[50]** | NA | NA | NA | | Bacterial filamentous colonies of actinomycosis surrounded by a suppurative reaction with areas of necrosis | NA | NA | NA | HE |
| **Turkey, 2002**  **[51]** | NA | NA | NA | | Sulfur granules and filamentous organisms consistent with an actinomycotic abscess | NA | NA | NA | HE |
| **Turkey, 2003**  **[52]** | NA | Not diagnostic | NA | | NA | Microscopic examination of material spontaneously drained from the pustules |  | Positive of drained from the pustules | culture |
| **Turkey, 2006**  **[53]** | NA | NA | NA | | Chronic inflammation showing abscess formation containing actinomyces colonies | NA | NA | Negative | HE |
| **Turkey, 2007**  **[54]** | NA | Sulfur granules surrounded by neutrophil leucocytes | NA | | NA | NA | NA | Negative | HE |
| **Turkey, 2007**  **[55]** | NA | Acute and chronic inflammation in continuation with liver parenchyma, granulation tissue, and gram-positive branching filamentous rods consistent with an Actinomyces species | NA | | NA | NA | NA | NA | HE |
| **UK, 2009**  **[2]** | culture of hepatic mass aspirate | NA | NA | | NA | NA | NA | Positive of liver abscess drainage | Culture |
| **UK, 2011**  **[56]** | NA | NA | NA | | NA | NA | abscesses centered around Actinomyces colonies. The liver parenchyma was replaced by fibrosis and granulomatous inflammation. | NA | HE |
| **UK, 2012**  **[57]** | culture of hepatic mass aspirate | collections of eosinophilic granules consistent with actinomyces-induced abscess formation | NA | | NA | Pipelle endometrial biopsy showed centrally branching filaments representing actinomyces. | NA | Positive from liver drainage | HE and culture |
| **USA, 2000**  **[58]** | Cultures of the material collected on ultrasound guided biopsy of the liver lesion revealed only *Peptostreptococcus* | NA | NA | | NA | Gram stains of the smears of the pleural fluid aspirate revealed microorganisms consistent with Actinomyces | NA | Negative | Gram staining |
| **USA, 2001**  **[59]** | NA | Not diagnostic | NA | | Branching, filamentous, Gram-positive bacteria with a sulfur granule appearance consistent with actinomycoses were readily apparent in both the kidney and liver biopsy specimens. | NA | NA | Positive from liver biopsy | HE and culture |
| **USA, 2002**  **[60]** | NA | NA | NA | | NA | NA | NA | Positive from liver absecces drained  Positive blood culture | culture and PCR |
| **USA, 2002**  **[61]** | Gram staining revealed numerous Gram-positive branching filamentous rods consistent with Actinomyces species | NA | NA | | NA | NA | NA | Negative | Gram staining |
| **USA, 2005**  **[62]** | NA | NA | NA | | NA | CT-guided transthoracic needle aspiration revealed purulent material. A Gram stain of the fluid showed many white blood cells and Gram-positive, filamentous, branching organisms. | NA | Negative | Gram staining |
| **USA, 2006**  **[5]** | Culture of hepatic mass aspirate | NA | NA | | NA | NA | NA | Positive from liver absecces drainage | Culture |
| **USA, 2006**  **[5]** | Culture of hepatic mass aspirate | NA | NA | | NA | NA | NA | Positive from liver absecces drainage | Culture |
| **USA, 2010**  **[10]** |  | Not diagnostic |  | | Neutrophilic micro abscesses containing gram-positive, filamentous, radially oriented bacteria in Brown and Brenn stained sections, consistent with actinomycosis |  |  | NA | HE |
| **USA, 2011**  **[63]** | NA | NA | Inflammatory granulation tissue and granules consistent with hepatic actinomycosis | | NA | NA | NA | Positive from liver resection | HE and culture |
| **USA, 2013**  **[64]** | culture of hepatic mass aspirate | NA | NA | | NA | NA | NA | Positive from liver absecces drainage | Culture |
| **USA, 2016**  **[65]** | NA | NA | classical Sulphur granules of Actinomycetes | | NA | NA | NA | Negative | HE |
| **USA, 2016**  **[66]** | NA | Numerous neutrophils, vaguely formed granulomas, multinucleated giant cells, bile pigment, hepatocytes and a few tangled clumps of filamentous microorganisms, resembling ‘cotton balls’. | NA | | NA | NA | NA | Negative | HE |
| **USA, 2017**  **[67]** | NA | Colony with a sulfur granule in relation to hepatic parenchyma, associated inflammation, and fibrotic response: Splendore–Hoeppli phenomenon | NA | | NA | NA | NA | Negative | HE |
| **USA, 2017**  **[68]** | Abundant  neutrophils and gram positive rods | Negative for any malignancy. | NA | | NA | NA | NA | Positive from liver drainage | HE and culture |
| **USA, 2019**  **[69]** |  | The aspirate was negative for malignancy and revealed the presence of Gram-positive filamentous bacteria | NA | | NA | NA | NA | NA | HE |
| **USA, 2020**  **[70]** | NA | not diagnostic | not diagnostic | | NA | NA | NA | Negative | PCR |

NA: not applicable

**Tables S2:** Radiological and anatomic characteristics of hepatic actinomycosis*.*

| **Country, year of publication and references** | **CT** | **Abdominal ultrasonography** | **MRI** | **Invasion and complications** |
| --- | --- | --- | --- | --- |
| **USA, 2020**  **[70]** | Numerous, approximately 8, hypo attenuating lesions within both hepatic lobes | Confirmed both complex cystic and mixed cystic/solid lesions within the liver | NR | NR |
| **USA, 2019**  **[69]** | A hepatic lesion with heterogeneous enhancement measuring 5.634.2 cm. | NR | Repeat cross sectional imaging with Eovist-enhanced MRI showed a dramatic decrease in size of the lesion seen at presentation, measuring 3.4 3 1.6 cm | NR |
| **Serbia, 2018**  **[43]** | Multinodular abscess (12 cm in diameter), located in the right lobe of the liver, between segments 4 and 8, with infiltration of the anterior abdominal wall | Multilocular abscess (12 cm in diameter), located in the right lobe of the liver, between segments 4 and 8, with infiltration of the anterior abdominal wall | NR | Infiltration of the anterior abdominal wall |
| **Korea, 2018**  **[38]** | Abscess in the left lower quadrant, a hepatic lesion and bilateral hydronephrosis | Ultrasound revealed an intra-uterine device (IUD | NR | NR |
| **Korea, 2018**  **[39]** | An 8.5 7.8-cm ill-defined suspicious, delayed enhancing, lobulated mass-like lesion in the right hepatic lobe with hyperemic change of adjacent hepatic parenchyma and segmental portal vein obliteration | NR | Ill-defined suspicious, delayed enhancing, lobulated mass-like lesion in the right hepatic lobe. | Liver mass invaded the right lower lung after wrong treatment |
| **USA, 2016**  **[65]** | 6.8 cm large liver abscess in the right lobe of the liver | NR | NR | NR |
| **China, 2016**  **[23]** | 6 × 6 cm hypodense mass in the left lateral segment of the liver | NR | NR | NR |
| **Korea, 2013**  **[8]** | Ill-defined hepatic masses with heterogeneous low attenuation on delayed phase. | NR | NR | Invaded into the muscle layer of right abdominal wall |
| **Portugal, 2014**  **[4]** | Segment IV of the liver a pseudonodularlesion, hypoechoic, heterogeneous with partially undefined limits with 45 × 33 mm and presenting low enhanced in the arterial phase, with pro-gressive enhanced in the late phase | In segment IV of the liver a pseudo nodular lesion, hypoechoic, heterogeneous with partially undefined limits with 45 × 33 mm | Nodular lesion with lobulated and undefined limits of 4 cm in its greatest diameter, hypointense signal on T1 and hyper intense signal onT2 | NR |
| **China, 2014**  **[12]** | 11.2 cm × 5.8 cm × 7.4 cm mass with an unclear edge in the left hepatic lobe, which involved Ⅱ, SⅡ, SⅢ and SIV. | NR | NR | NR |
| **Japan, 2014**  **[32]** | Multiple ring-enhancing solid masses in the right hepatic lobe with a maximum size of 4 cm in diameter | Lesions were vague and hypoechoic on B-mode ultrasonography, although they were clearly visualized in the Kupffer phase of perflubutane contrast-enhanced ultrasonography | NR | Direct infiltration to the Diaphragm and Thorax |
| **France, 2013**  **[24]** | Two liver abscesses and an enlarged appendix with a pseudotumoral appearance | NR | NR | NR |
| **China, 2013**  **[22]** | A hypodense lesion in the left lobe of the liver, with infiltration to the surrounding tissue | A mass in his left liver lobe that measured approximately seven cm in diameter | NR | Extended to the gastric muscularis propria |
| **Taiwan,2009**  **[1]** | Not showed any lesions in the liver due to little size | not showed any lesions in the liver | No | NR |
| **Korea, 2012**  **[35]** | Several lesions of low-attenuation in both lobes of the liver | NR | NR | NR |
| **Korea, 2012**  **[36]** | Hepatic and ovarian masses | NR | Multiple large hepatic masses in the right lobe of the liver (the diameter of the largest hepatic mass was 12.3 cm) with peritoneal metastases | NR |
| **Spain, 2011**  **[44]** | A large, multiloculated lesion in the left hepatic lobe with hypodense areas suggestive of cystic or necrotic degenerative tissue | NR |  | Cardiac tamponade complicating primary hepatic actinomycosis |
| **Japan, 2012**  **[9]** | Multiple solid lesions with ring enhancement in the right hepatic lobe, and cystic lesions with septa in the left hepatic lobe and the right infra diaphragm | NR | Hepatic lesions with a decreased signal intensity on T1-weighted and T2-weighted images in the central area | Acute cardiac tamponade caused by the extension of hepatic abscess |
| **USA, 2011**  **[63]** | Non-specific abnormality in the right lobe of the liver, suspicious for a mass. | NR | Was positive for a mass, suspicious for an isolated tumor recurrence between segments 5 and 6 in the liver | NR |
| **UK, 2011**  **[56]** | NR | NR | NR | NR |
| **China, 2011**  **[21]** | A mass with mixed solid and cystic components in the left lateral segment | NR | T2-weighted magnetic resonance images, inflammatory pseudotumor with dense fibrosis deposition is usually represented as isointensity or hypointensity,2,5 whereas other diseases are often represented as hyperintensity.4 | Hepatic lesion directly invaded the diaphragm |
| **Japan, 2011**  **[31]** | A low-density cystic lesion on the surface of S4 of the liver, and low to iso-density fluid collection was found around the lesion | Low-echoic mass lesion (4×9 cm) on the surface of S4 of the liver | NR | NR |
| **Turkey 2010**  **[50]** | Showed hypodense cystic lesions without prominent contrast enhancement. | Abdominal ultrasound revealed two large heterogeneous masses involving the posterior segment of the right lobe of the liver and one heterogeneous mass on the medial segment of the left lobe. | NR | NR |
| **UK, 2009**  **[2]** | A 5.7cm in diameter hypodense lesion in the right lobe of the liver, suggestive of a liver abscess | NR | NR | NR |
| **Greece, 2010**  **[28]**  **26** | Multiple hepatic hypodense solid lesions in both hepatic lobes | NR | NR | NR |
| **USA, 2010**  **[10]** | A lesion in the posterior right lobe of the liver near the dome, measuring 6.9 × 7.4 cm | A lesion in the posterior right lobe of the liver near the dome, measuring 6.9 × 7.4 cm | NR | Nodules were visualized at the lung bases |
| **Turkey, 2007**  **[54]** | A hypodense and abscess-like mass with contrast enhancement of 3.5×3.5×2.5 cm on segment 6 | A hypoechoic lesion of 3.5×3.5 cm in the posterior inferior region of the right lobe of the liver | NR | NR |
| **Kore, 2006**  **[34]** | A lesion with reduced attenuation in the right hepatic lobe | A 5.5 cm sized mass that looked like a metastatic lesion in the right hepatic lobe. | NR | NR |
| **Turkey, 2006**  **[53]** | Two hypodense subcapsular lesions with thick walls and enhanced contrast in the wall in post contrast series in the posterior segment of the right liver lobe | Two hypoechoic lesions with heterogeneous internal structure, subcapsular localization and regular contours in the posterior segment of the right liver lobe. | NR | Intensive pleural effusion with air-fluid levels and accompanying compression atelectasia. |
| **Italy, 2005**  **[30]** | 3 heterogeneous and hypodense focal lesion in the IV hepatic segment measuring 5 cm in diameter | a heterogeneous and hypodense focal lesion in the IV hepatic segment measuring 5 cm in diameter | NR | NR |
| **USA, 2005**  **[62]** | A complex, septated fluid collection with a thick, irregular, contrast enhancement in the wall involving the liver and extending into the posterior para renal space | NR | NR | Infiltration of the diaphragm and right lung |
| **India, 2005**  **[29]** | Three hypodense coalescing lesions in the right lobe of the liver close to the surface and measuring 10 cm×5 cm when taken together | NR | NR | Track extending from the liver abscess into the abdominal wall. |
| **China, 2004**  **[20]** | A hypodense mass measuring 6 cm in diameter in the left lateral segment of the liver | A mass in the left lobe of the liver that measured about 6 cm | NR | Abscess had involved the stomach with extension to the gastric muscularis propria resulting in focal fibrosis and inflammation. |
| **Canada, 2005**  **[19]** | A low density hepatic mass | NR | NR | NR |
| **Greece, 2004**  **[27]** | A 10 /8 cm hypoechoic solid mass in the right liver lobe. | A 10 /8 cm hypoechoic solid mass in the right liver lobe. | NR | NR |
| **Turkey, 2003**  **[52]** | A lesion within the left lobe of the liver and invasion of anterior abdominal wall. | A lesion within the left lobe of the liver and invasion of anterior abdominal wall. | NR | Fistulization through the abdominal wall and spontaneous draining from the skin |
| **Turkey, 2002**  **[51]** | NR | A mass, containing image of fluid, in the right upper quadrant, consistent with the region of the hepatic colon flexura. | NR | A mass extending from hepatic flexura into the transverse colon |
| **USA, 2002**  **[60]** | NR | NR | A cystic mass in the dome of the right lobe of the liver | hepatic abscess was presumed to be the cause of the patient’s Thrombotic Thrombocytopenic Purpura (TTP) |
| **USA, 2002**  **[61]** | A 7.5*6.0 cm2 lesion in the liver with central necrosis and fluid extending along the posterior medial segment of the right lobe of the liver | NR | NR | Large liver abscess extending to the capsule, diaphragm and the space between the liver and right kidney |
| **Germany,2001**  **[26]** | Abscess formation in segments 4 and 8 | NR | NR | NR |
| **Canada, 2001**  **[18]** | A solitary hepatic mass saddling segment 4, 8 and 5, and measuring 9 cm in its largest diameter | NR | NR | Pulmonary infiltrate in the right lower lobe |
| **Taiwan, 2001**  **[46]** | Several thickened shadows in the posterior segment of the side of the liver | ‘‘tumor mass’’ over the upper pole of the right kidney. | NR | Liver abscess extending to Gerota’s fascia with encasement of the upper pole of the right kidney and severe inflammation between the hepatic flexure of the transverse colon the liver bed |
| **USA, 2000**  **[58]** | Several hypodense lesions in the liver suspicious for either abscess or tumor. | NR | NR | NR |
| **Bahrain, 2020**  **[17]** | A large heterogeneous irregular hepatic mass with intrahepatic biliary duct dilatation. | Multiple hypoechoic lesions in the liver | NR | NR |
| **USA, 2017**  **[67]** | NR | NR | 3 ill-defined large liver masses, the largest measuring 6.3 4.6 cm | NR |
| **USA, 2017**  **[68]** | Septated sub pulmonic mass measuring 5.6 cm × 2.2 cm with internal high density anterior to the liver and adjacent to the diaphragm and 3.7cm × 2.2 cm fluid collection posterior to the anterior abdominal wall musculature in the RUQ extending to the right rectus muscle | NR | NR | NR |
| **Spain, 2017**  **[45]** | NR | Cholelithiasis with no signs of cholecystitis or dilatation of the intrahepatic biliary tract | An abscess was observed in hepatic segment V, measuring 4.4 cm 5 cm | NR |
| **USA, 2016**  **[66]** | An exophytic, enhancing liver lesion of the right hepatic lobe with surrounding infiltrates of uncertain etiology | NR | NR | Inflammatory changes of the right abdominal wall, right iliopsoas muscle and quadratus lumborum |
| **Japan, 2016**  **[33]** | Multiple liver masses | Multiple liver masses | NR | Intravenous thrombosis of the left hepatic vein extending into the inferior vena cava |
| **Taiwan, 2013**  **[49]** | A huge, heterogeneous, mixed solid and cystic mass occupying nearly the entire right hepatic lobe | NR | NR | NR |
| **USA, 2013**  **[64]** | Two large irregular hypodense masses within the liver and irregular circumferential bowel wall thickening of the caecum | An ill-defined mass lesion in the superior aspect of the right hepatic lobe measuring 4.6×4.1×4.1 cm | NR | NR |
| **Romania, 2013**  **[41]** | Confirmed the linear, spontaneously hyper dense image in the posterior gastric wall, as well as the liver mass | A solid, ill circumscribed liver tumor, situated in the left lobe of the liver, measuring about 70/60 mm | NR | Hepatic artery invasion, encasement of the portal vein and celiac and liver hilum adenopathies |
| **Romania, 2012**  **[40]** | A hypodense mass of 12.4 x 10.5 cm located in the seventh segment of the right liver lobe | One heterogeneous mass of 10.5 x 8.5 cm in the right liver lobe. | NR | NR |
| **UK, 2012**  **[57]** | A 2.4 cm irregular low-attenuation density area was now noted in the right lobe of the liver. | NR | NR | NR |
| **Korea, 2012**  **[37]** | Multiple micro abscesses were found in the liver and lung | NR | NR | NR |
| **Taiwan, 2010**  **[48]** | One heterogeneous echogenic mass lesion, 5.7 9 4.0 cm, over the left lobe of the liver | One heterogeneous echogenic mass lesion, 5.7 9 4.0 cm, over the left lobe of the liver | NR | NR |
| **Serbia, 2009**  **[42]** | Several liver lesions in the right lobe, the largest measuring 6.7 × 6.8 cm, 5.7 × 3.2 cm and 3.2 × 2.7 cm | Several liver lesions in the right lobe, the largest measuring 6.7 × 6.8 cm, 5.7 × 3.2 cm and 3.2 × 2.7 cm | NR | Propagation towards diaphragm with pericardial and right pleural effusion in our case |
| **Germany, 2009**  **[25]** | NR | A large hypodense mass (6 × 6.5 cm) within the right liver lobe and pathologically enlarged lymph nodes in the peri portal region | An irregularly shaped, inhomogeneous and hypointense lesion of the right liver lobe (6 × 8cm in segments 7 and 8) with multiple satellite lesions | NR |
| **Turkey, 2007**  **[55]** | Diffuse fatty infiltration of the liver and a solid mass of 6 9 5 cm located in the fourth segment of the left liver lobe | A hyperechoic mass of 6 9 5 cm in the left liver lobe | NR | NR |
| **Taiwan, 2005**  **[47]** | Identified two hypodense lesions with septum and peripheral ring enhancement patterns on the same sites | Two hypoechoic lesions over bilateral hepatic lobes (2.1 3.1 cm at segment 2 and 1.8 3.8 cm at segment 7) | NR | NR |
| **Taiwan, 2005**  **[47]** | A hypodense mass (5.2 7.0 cm) with peripheral enhancement in the same area | A 6-cm isoechoic hepatic tumours with hypoechoic ring at right posterior inferior segment | NR | NR |
| **USA, 2001**  **[59]** | A right upper pole renal mass, possible contiguous spread to the right lobe of the liver , probable lymph node involvement, a large mass in the left lobe of the liver | Right renal mass was identified | NR | NR |
| **USA, 2006**  **[5]** | Multiple foci of pulmonary emboli, bilateral consolidations, a pericardial effusion, and several low-density lesions in the liver, the largest within the caudate measuring | NR | NR | Hepatic vein and IVC thromboses hypothesize an Actinomyces-infected clot as the source of septic pulmonary emboli |
| **USA, 2006**  **[5]** | Right anterior subphrenic and subcapsular fluid collections. The right anterior subphrenic collection showed higher attenuation than simple fluid, suggesting exudate or hemorrhage. | NR | NR | NR |

CT: Computed Tomography. MRI: Magnetic Resonance Imaging. NR: not reported.

**Table S3:** Prophylaxis and Empirical antibiotics therapy in patient with hepatic actinomycosis.

| Country, year and reference | Empirical treatment | Outcome | Final treatment |
| --- | --- | --- | --- |
| Japan, 2014  [32] | Intravenous imipenem/cilastatin | Fever did not improve and the lesions had not liquefied one month after the induction of treatment | A drainage tube was inserted into the right thoracic space and Ampicillin/sulbactam |
| France, 2013  [24] | Amoxicillin clavulanate 200 mg BID, ofloxacin 500 mg TID, and metronidazole 500 mg TID | Despite ongoing antibiotic therapy, the patient remained febrile at 38-38.5◦C with persistence at the 10th postoperative day of poor general condition and laboratory signs of inflammation. | Appendectomy and ampicillin |
| Taiwan,2009  [1] | 1 g ceftriaxone every 12 hours and 500 mg metronidazole every 8 hours | High fever persisted for 6 days of treatment. | Resected spleen and liver nodules and penicillin |
| Spain, 2011  [44] | Imipenem–cilastatin (500 mg, four times daily), amikacin (1 g, once daily), and linezolid (600 mg, twice daily). | Patient had become afebrile |  |
| Japan, 2012  [9] | Sulbactam/cefoperazone and gentamycin | Cardiac tamponade caused by  the extension of hepatic abscess | Video-assisted thoracic surgery and Sulbactam/ampicillin |
| Japan, 2011  [31] | Intravenous cefmetazole, 2 g every 12  hours, was started after drainage then changed from cefmetazole to trimethoprim/sulfamethoxazole (800/160 mg orally once a day) and ampicillin/sulbactam (2/ 1 g intravenously every 24 hours) on the assumption of actinomycosis, nocardiosis and infection of other multiple microorganisms. | Not reported | Aspiration drainage and placed an indwelling catheter immediately, ampicillin and Amoxicillin |
| USA, 2010  [10] | Ciprofloxacin and flagyl for treatment of a potential abscess | Not reported | Clindamycin |
| India, 2005  [29] | Tinidazole 800 mg per day and cefotaxime 1 g twice a day i.v. along with paracetamol, antacids and vitamins. | The patient continued to have spikes of fever and pain in the abdomen; tenderness increased in the right hypochondrium, with bulging of the abdominal wall. | Ultrasound-guided percutaneous aspiration of  the liver abscess, penicillin and ampicillin |
| Greece, 2004  [27] | Intravenous ciprofloxacin (600 mg t.i.d.), clindamycin (600 mg t.i.d.) and vancomycin (1 g b.i.d.) was continued for 20 day | Symptoms and liver ultrasound findings remained unchanged. | A right posterior segmentectomy of the liver was performed and ciprofloxacin |
| Japan, 2016  [33] | Intravenous cefozopran followed by intravenous meropenem infusion | No improvement | Right salpingo-oophorectomy and levofloxacin |
| USA, 2013  [64] | Levofloxacin and piperacillin-tazobactam | The patient continued to be febrile over the following 36 h | Drainage catheter and amoxicillin-clavulanate |
| Romania, 2012  [40] | Meropenem 3 g | No improvement | Ampicillin and Doxycycline |
| Taiwan, 2010  [48] | Ceftriaxone (1 g every 12 h) | The condition of the patient improved with this medical treatment and with CT-guided pigtail catheter insertion for drainage of the abscess | Ampicillin–sulbactam and Amoxicillin |
| Taiwan, 2005  [47] | Cefazolin and gentamicin | Although fever and pain were subsided following antibiotic treatment, the tumor expanded to 8 cm in diameter 3 months later | Penicillin V 2 g per day for 3 months postoperatively |
| USA, 2006  [5] | Ceftriaxone and Doxycycline were started empirically | Bilateral upper lobe pneumonias resulted in a worsening of the patient’s respiratory status, requiring ICU admission and intubation | Percutaneous aspiration of the two largest liver collections and penicillin |
| USA, 2006  [5] | Metronidazole and levofloxacin | No improvement | Drainage and penicillin |

**Table S4:** Microbiological features of hepatic actinomycosis.

| Country, year and reference | Companion bacteria | Isolation source | Description |
| --- | --- | --- | --- |
| **USA, 2016**  **[65]** | *Escherichia coli* | Resected liver | These concomitant  infections are known to enhance the pathogenicity of *Actinomycetes* |
| **Spain, 2011**  **[44]** | *Fusobacterium* spp. and *Peptostreptococcus* | Pericardial fluid |  |
| **Japan, 2012**  **[9]** | *Klebsiella oxyyoca and Citrobacter*  *koseri* | Cardiac effusion | Both aerobic and anaerobic bacterial cultures from liver biopsy were negative |
| **Kore, 2006**  **[34]** | Single colony of coagulase negative  *Staphylococcus* | Various tissue samples obtained from surgery | Cultures of pus and tissues gained by surgical intervention did not reveal *Actinomyces*  species |
| **China, 2004**  **[20]** | *Streptococcus milleri* | Liver mass | Bacterial culture taken from the liver mass only grew this bacterium |
| **Canada, 2005**  **[19]** | *Escherichia coli* and *Klebsiella* species (from urine)  *Fusobacterium nucleatum* and Gram-negative rods. | Urine and blood | Liver biopsy culture confirmed *Actinomyces* species infection |
| **Greece, 2004**  **[27]** | non-A, non-B hemolytic *streptococcus* grew in multiple | Blood |  |
| **USA, 2002**  **[60]** | *Bacteroides fragilis* | Blood | The aspirate obtained from the hepatic abscess yielded *A. turicensis,* and cultures  of blood samples obtained after admission yielded *A. turicensis* and *Bacteroides fragilis.* |
| **USA, 2002**  **[61]** | *Proteus mirabilis* | Aspiration of the hepatic lesion | Aerobic culture grew Proteus mirabilis but anaerobic  culture was negative. |
| **Germany,2001**  **[26]** | *Prevotella oris* | Liver aspirate | Analysis of amplified nucleic acid within the aspirate revealed this bacterium and after this finding antibiotic was changed from penicillin to Clindamycin. |
| **USA, 2000**  **[58]** | *Peptostreptococcus* | Material collected on ultrasound guided  biopsy of the liver lesion | Negative blood culture |
| **Spain, 2017**  **[45]** | *Streptococcus anginosus* and *Staphylococcus epidermidis* | Percutaneous drainage of the liver abscess |  |
| **USA, 2013**  **[64]** | *Eikenella corrodens* | Percutaneous drainage of the liver abscess | *Candida albicans* was also  isolated, but patient improvement without antifungal treatment |
| **UK, 2012**  **[57]** | Mixed anaerobes as well as *Actinomyces israelii* | Percutaneous drainage of the liver abscess |  |
| **Germany, 2009**  **[25]** | Anaerobic bacteria and *Klebsiella pneumonia* | Liver biopsy | *Klebsiella pneumonia* resistant to Amoxicillin, which were most likely selected during long-term  treatment with Amoxicillin |
| **Taiwan, 2005**  **[47]** | *Pseudomonas aeruginosa* | Liver biopsy | Negative blood culture |
| **USA, 2001**  **[59]** | *Actinobacillus actinomycetemycomitans* | Liver biopsy |  |
